# FAMILY CAREGIVER CONTRIBUTIONS TO HOME HEALTH FOR OLDER ADULTS: PREDICTORS OF ADEQUATE SUPPORT

**DOI:** 10.1093/geroni/igz038.748

**Published:** 2019-11-08

**Authors:** Julia Burgdorf, Julia Burgdorf, Judith D Kasper, Jennifer L Wolff

**Affiliations:** Johns Hopkins Bloomberg School of Public Health, Baltimore, Maryland, United States

## Abstract

Family caregivers play a crucial role in supporting older adults through home health (HH) episodes; yet no prior research examines factors affecting the adequacy of caregiver support during HH. We use a novel dataset linking nationally-representative survey data with HH assessment data for community-dwelling older adults (n=2,128) to identify older adult characteristics associated with adequate caregiver support in four task categories (Activities of Daily Living, medication management, medical tasks, and safety/oversight) during a subsequent HH episode. Weighted, multivariable logistic regression is used to model the likelihood of adequate caregiver support in each category. Multiple older adult characteristics prior to the HH episode were associated with greater likelihood of adequate caregiver support during HH, including: prior caregiver assistance with mobility, self-care, and health care tasks, living with others, and cognitive impairment. These findings reveal new risk factors for consideration in risk assessment and payment adjustments related to social determinants of health.

